# A Case Study on EEG Signal Correlation Towards Potential Epileptic Foci Triangulation

**DOI:** 10.3390/s24248116

**Published:** 2024-12-19

**Authors:** Theodor Doll, Thomas Stieglitz, Anna Sophie Heumann, Daniel K. Wójcik

**Affiliations:** 1 Biomaterial Engineering, Hannover Medical School, 30625 Hannover, Germany; 2 Laboratory for Biomedical Microtechnology, Department of Microsystems Engineering (IMTEK) and BrainLinks-BrainTools Center, University of Freiburg, 79085 Freiburg im Breisgau, Germany; thomas.stieglitz@imtek.uni-freiburg.de; 3 Nencki Institute of Experimental Biology of the Polish Academy of Sciences, 02-093 Warsaw, Poland; d.wojcik@nencki.edu.pl

**Keywords:** clinical electric source imaging, signal propagation, time delay correlation, EEG, ECoG

## Abstract

The precise localization of epileptic foci with the help of EEG or iEEG signals is still a clinical challenge with current methodology, especially if the foci are not close to individual electrodes. On the research side, dipole reconstruction for focus localization is a topic of recent and current developments. Relatively low numbers of recording electrodes cause ill-posed and ill-conditioned problems in the inversion of lead-field matrices to calculate the focus location. Estimations instead of tissue conductivity measurements further deteriorate the precision of location tasks. In addition, time-resolved phase shifts are used to describe connectivity. We hypothesize that correlations over runtime approaches might be feasible to predict seizure foci with adequate precision. In a case study on EEG correlation in a healthy subject, we found repetitive periods of alternating high correlation in the short (20 ms) and long (300 ms) range. During these periods, a numerical determination of proportions of predominant latency and, newly established here, directionality is possible, which supports the identification of loops that, according to current opinion, manifest themselves in epileptic seizures. In the future, this latency and directionality analysis could support focus localization via dipole reconstruction using new triangulation calculations.

## 1. Introduction

The localization of one signal source (Electric Source Imaging [1,2], ESI) in the midst of a concert of multiple sources is one of the main challenges in brain research [3]. We currently have to rely on clinically established imaging techniques such as EEG, ECoG, PET, MEG, or fMRI data to detect single sources [4,5]. Dipole field measurements are still the subject of research [6].

In addition to fundamental knowledge-driven research, clinicians in epileptology and neurosurgery are in need of precise and reliable methods to localize seizure foci in patients with focal epilepsy [3], which are difficult to treat with medication [7], but can be made seizure-free by resection of the epileptogenic brain tissue [3,8] with good success. Since the probability of cognitive failures also increases with the size of the resected tissue [9], attempts are made to keep the resected tissue small. Therefore, it is essential to localize the focus precisely, usually by placing subdural ECoG electrode strips or arrays, which have a typical distance of 1 cm, prior to epilepsy surgery. These arrays achieve a spatial resolution of about 1 cm [10] due to their geometry, although millimeter-accurate spatial localization is reported in another source [11]. Recent studies using high-density microECoG arrays with only 1 mm electrode spacing have obtained increased precision in resolution [12]. However, they showed local phenomena such as mini-ictus but no clear increase in localization precision. As an alternative, several intracranial Stereo-EEG (SEEG) electrodes [13,14,15] are implanted but need prior knowledge about useful placement, which has to be obtained from non-invasive EEG studies. The typical interelectrode distance is on the order of 1 cm [16]; however, multiple designs are in progress to constantly improve spatial resolution. Reports from preclinical studies on animal models report dimensions in the order of tens of micrometers [17]. Imaging techniques like PET and fMRI also achieve localization precision at the centimeter level [18].

The localization of epileptic seizure foci uses mathematical analysis of EEG or ECoG signals to predict a dipole as the origin of the seizure based on the measured signals and the electrode location. Two major methods have been elaborated from completely different disciplines:

The first method is well-established for the analysis of the epicenter (i.e., the source) of earthquakes measured by structure-borne sound, which resembles ictal events in epilepsy by their shapes. In seismology, we have primary and secondary (near-surface) waves that propagate at 2–8 km/s and approximately 3 km/s, respectively. With the known data on the structure of the Earth’s crust regarding sound propagation, the epicenters can be determined with an accuracy of a few 10 km, given the Earth’s radius of 6400 km. If this procedure were applied to ictal events [19] with neuronal signal conduction velocities of 30 to 70 m/s and an actual maximum time resolution of EEG and ECoG data of 1 s, a spatial resolution between 3 cm and 7 cm could be achieved. This simplified assumption and today’s clinical standards in sampling rates (i.e., temporal resolution) would not accomplish the goal of high spatial accuracy in source localization.

In the second approach, it is assumed that the electrical field from the nerve cells propagates instantaneously at the speed of light [20]. As a result, source localization is not attempted via latencies, but via the calculation of dipole fields, which are reconstructed from the signals recorded by EEG and ECoG [21,22] and the knowledge of the position of the electrode sites. The more electrode sites are placed, the more precise the dipole field can be reconstructed.

The tendency for high-channel count electrode arrays with small electrodes diameters and high integration densities [23] has been transferred into clinical research [24]. High-resolution EEG or intracranial iEEG is taken for an initial search for the ictal focus via the nearest and overlying electrode with the help of deep neural networks (DNN) for the detection of the earliest discharge and support vector machines (SVM)-assisted filters and classifiers [25,26].

In clinical practice, localization is performed by the various methods of electromagnetic source identification (ESI) from scalp EEG and depth electrode recordings (iEEG), which are combined with other imaging techniques such as MRI, PET, and single-photon emission computed tomography (SPECT). The well-known ESI methods dynamic statistical parametric mapping (dSPM) and weighted minimum norm estimate (wMNE) [27], as well as standardized low resolution brain electromagnetic tomography (sLORETA) [28] and standard maximum entropy on the mean (cMEM) [29], solve the inverse problem for reconstructing dipole fields in the brain as a cause of electrical potentials recorded from electrodes on the skull or on the brain. More recent versions of ESI are based on kernel ESI and gradient-free kernel Current Source Density (kCSD) [30], which calculate multiple sources for arbitrary electrode distributions while taking anatomical conductivity distributions into account. In addition to these approaches, calculations of connectivity in the brain from EEG and MEG are also used. Methods include (linear) cross-correlation, non-linear correlation, mutual information (MI), and phase-locking value (PLV) [31]. These calculations are primarily used to identify temporal network interactions of multiple sources [32]. Both the inverse solution and the connectivity analysis have been shown to be powerful not only in the interictal phases but also during seizures, where they show a strong increase in synchronicity [33,34,35]. In the recent past, however, hardly any numerical delays between signals from different electrode positions have been used for source localization.

Further approaches include the application of wavelet entropy and sliding window techniques [36], as well as Kalman filtering [1]. Moreover, electric stimuli are applied to either elicit an ictus for the localization of the seizure onset zone [37] or to suppress epileptic seizures [38], which can be used as a treatment option.

Apart from the noise sensitivity of inverse modeling, all approaches have limitations [39] in identifying the underlying causes of the seizure. These may lie at the level of singular neurons, where the self-inhibition mechanisms of the ion channels may be suspended, or at the network level of groups of pyramidal or subpyramidal-pyramidal clusters [40,41]. Alterations may occur at the level of electrical synapses or in the course of neuronal plasticity [42] of an epileptic predisposition of structures which also build recurrent signaling pathways [43] that exhibit an inherent tendency to giant postsynaptic potentials or overshoots [44]. The latter two conditions have anatomical-structural prerequisites that would favor direct neuronal latency analysis. However, the overall picture would, in any case, be a superimposition of delayed neuronal signal transmissions with instantaneous field propagation and attenuation in magnitude by glial tissue, for example.

This work addresses the question of to what extent delayed neural signals could be useful for localizing epileptic foci. The first step will be the investigation into whether the same relevant time delays can be derived from recordings at several measuring points. Correlation analysis will be the method of choice. In the second step, potential directionality of the signals needs to be derived from the correlation results to identify if the recording electrodes are closer to or further away from an assumed source.

These two fundamental objectives do not need any epileptic activity for fundamental analysis. Therefore, EEG signals from a healthy subject were acquired. With respect to epileptic seizure detection, the following assumptions were made: the occurrence of characteristic ictal frequencies in the EEG requires either steady-state loops with positive feedback that exaggerate like feedback-howling or a local overexcitation that falls into exhaustion and back again. If these loops contributed to ictal EEG, they should still be present as a fundamental pattern in healthy states, albeit at a physiologically weaker level. In normal EEG, one should therefore find a directional dependence of the signals across the channels.

Such simple linear models would, in any case, fall short, as the continuous reorganization of the central connection loops must be taken into account. In clinical applications, successful runtime localization of foci would therefore at least require that short-term stable mappings of delays can be established, from which focus triangulation could be achieved in ictal cases. We hypothesize that these delay mappings would continuously reorganize or fluctuate dynamically and, at best, still fit the following ictus. Leaving aside the possibility of pre-ictal, visibly manifesting loops at first glance, subliminal, temporary delay mappings should also be discernible in healthy EEG.

To investigate our hypothesis, we first examine the time scales of the expected signals in detail, check the presence of distinct correlation periods, and finally look into their directionality.

## 2. Temporal and Spatial Design Considerations

The mathematical-physical model of delay-based epileptic excitation requires feedback loops with characteristic loop propagation times, τ_L_. These can range from short-range temporal lobe epilepsy, in which localization is often successful with conventional electrode systems and centimeter-accurate resolution, or τ_L_ ≈ 0.15 ms [12]. In extreme cases, long-range excitation loops are conceivable that run over a commissure and back, with path lengths of 2 × 14 cm = 28 cm and loop propagation times of around 8 ms, adding delays per synapse between 0.5 and 2 ms [45], and possible synaptic summation periods [46,47] on the same scale. In total, loop propagation times, τ_L_, of 5 ms up to more than 10 ms are conceivable here. These times reflect well the usual ictal frequencies of up to 200 Hz. The latter also sheds light on the possibility of local self-excitation considered above. Since the refractory period of nerve cells ranges between 1.5 ms and 2 ms, such a focus would oscillate at a frequency of at least 500 Hz. If lower frequencies occur, there is the need for a small loop to increase runtimes to obtain these frequencies in positive feedback loops.

**Figure 1:**
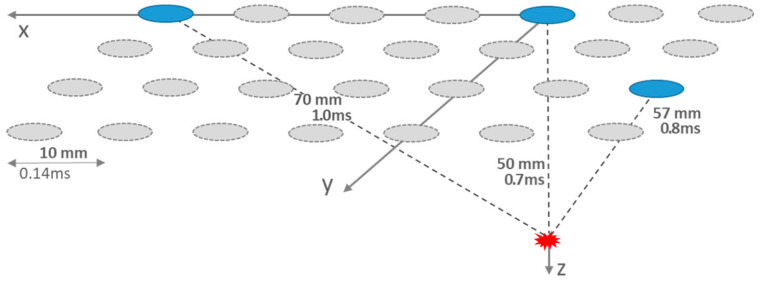
Surface (x,y-) triangulation scheme of an ictal source in the depth (z) of about 50 mm with electrodes of 10 mm pitch (spacing center to center). Neural propagation leads to different transit times of 0.7 ms, 0.8 ms, and 1.0 ms, respectively.

The conversion of the transit time differences to a location in space is obtained by triangulation of three points on the (skull/brain) surface toward their specific point of intersection (Figure 1). These points are placed in the centers at the electrodes and radii that result from the products of transit time differences and velocity of propagation [48]. As an example (Figure 1), a source depth of z = 50 mm under an electrode within an equidistant electrode array with 10 mm pitch (spacing center to center) at the origin of a Cartesian x,y,z coordinate system and two further electrodes, which were assumed to be five positions further in the x-direction and two diagonal positions. The calculated transit time differences were 0.1 ms and 0.3 ms [43]. Consequently, the temporal resolution limit should be at least 0.1 ms for centimeter-precise localization [44], which relates to a resolution in the frequency range of 10 kHz or, for sampling according to the Nyquist condition, 20 kHz. This comes up against the technical limits of today’s clinical electrophysiology setups.

Such approaches have been proposed for demultiplexing of ECoG signals [49], in support vector machines [50] to decode motor signals for BCIs [51], and to detect brain-heart disorders in focal epilepsies [52]. However, the idea that signal transmission via neurons could be visible in EEG is still controversially discussed based on the arguments regarding how much grey and white matter contribute [53].

## 3. Experimental Results

As elaborated in the introduction, a fundamental analysis of correlation periods does not need data from epilepsy patients. Therefore, data acquisition was conducted in the form of a case study by one of the co-authors in a self-experiment. At the time of data collection, the person was generally in good health, of sound mind, had no known (neurological) preexisting conditions, and generally has a good level of fitness in terms of sports physiology. The data collection was carried out while the person was awake and with his eyes open.

A 64-channel high-density EEG from BRAIN PRODUCTS was used. Using BRAINVISION professional ANALYZER2.1^®^ software, the impedance of the electrodes on the coupled EEG cap was checked to determine if it was below the usual standard of 15 kOhm at 1 kHz. The data collection took place in a lit anechoic room with playback of acoustic stimuli in a task to press a button under certain conditions as a response. Data from four runs of seven minutes each were recorded. The following data analysis uses only non-event-related sections. The data were recorded at 1 kHz and were down-sampled to 100 Hz for further latency analysis.

The determination of the correlation r of two data sets from channels p and q, shifted by a temporal latency Δ, is carried out according to a modified Bravais–Pearson, Equation (1),

(1) $$r_{p,q}\left(∆\right)=\frac{\frac{1}{n}\sum_{i=1}^{n}\left(p_{i}-\bar{p}\right)\left(q_{i+∆}-\bar{q}\right)}{\sqrt{{\frac{1}{n}\sum_{i=1}^{n}\left(p_{i}-\bar{p}\right)}^{2}}\cdot \sqrt{{\frac{1}{n}\sum_{i=1}^{n}\left(q_{i+∆}-\bar{q}\right)}^{2}}}$$

by direct comparison of the respective value pairs p_i_ and q_i+Δ_ of EEG amplitudes. The calculated correlation coefficients r_p,q_(Δ) can assume values between +1 and −1. A value of +1 occurs if the delayed signals q_i+Δ_ directly repeat the preceding signals p. Correlation coefficients around zero indicate incoherence, and values approaching −1 correspond to an anti-phase situation where the q_i+Δ_ data set is almost the negative of the preceding p series. A total of 8 min 50 sec recording time was examined per channel pairing for preset latencies of 10, 20, 30, 50, 70, 100, 200, 250, 300, 400, and 500 ms.

**Figure 2:**
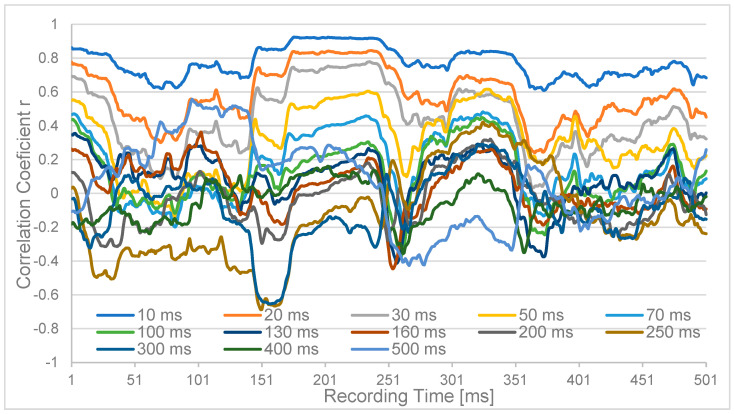
Correlation coefficient r of channels Oz and PO3 within a 500 msec correlation interval. The x-axis denotes Oz time over 500 ms, against which PO3 was delayed by 10 ms up to 500 ms. For the shorter delays, a positive correlation is always found, which tends to zero around 100 ms and turns into partial anticorrelation for delays <250 ms.

The results were time-resolved progressions of 500 msec (p-) duration (for clarity, we are using “ms” for delay times and the outdated units “sec” and “msec” to denote recording times) each (Figure 2) for the monopolar channels Oz and PO3 for the different latencies. While the short latencies up to 30 ms are consistently positively correlated, the longer latencies up to 300 ms fluctuate around zero, and the very long latencies tend to be anticorrelated.

We chose 34 EEG-recorded channels above one visual and auditory hemisphere and applied the correlation test over one full recording span of 8 min 50 sec. With one exception, all initial correlation coefficients reach almost +0.5. Eighteen out of thirty-four reached average r-values exceeding +0.7, corresponding to a coefficient of determination r^2^ of 49%. Table 1 provides an overview of the r-values determined for 10 ms latency. For highly correlated pairings, the delay r reaches +0.7. We observe that higher latencies tend to be shorter over the visual cortex than over the auditory areas.

**Figure 3:**
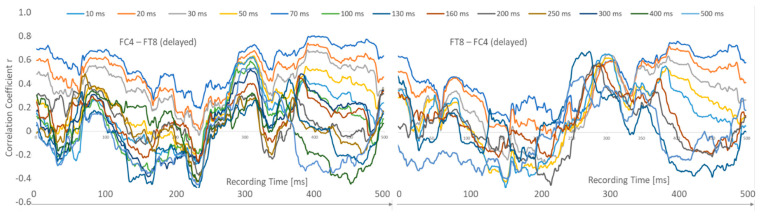
Correlation difference of the pairing FC4-FT8 versus FT8-FC4.

Next, the criterion of directionality was examined. Visual inspection of, for example, the neighbors afternext pairing FC4-FT8 (Figure 3) shows very similar 10 ms and 20 ms curves but different mean r-values during the first 270 msec recording period. In particular, the correlation curves for the 70 ms to 500 ms latencies are clearly different from one another. For the recording time 270–500 msec, the differences are significantly smaller, but the order of higher r-values is retained.

These observations were mathematically generalized and transformed into a numerical degree of directionality (Equation (2)). The correlation results p_i_, q_i+Δ_ and q_i_, p_i+Δ_ of two examined channels p and q are compared, and their maximum is ascertained as the best possible directionality measure as a percentage. At the same time, the latency at which this directionality measure is achieved is determined. The channel with the predominant source characteristic is then derived from the inner sign of the numerator in Equation (2): If the correlation of the signals p_i_, q_i+Δ_ shifted by Δ is greater than their counterpart p_i+Δ_, q_i_, p is more likely to be the source, and q_Δ_ repeats the signal.

(2) $$Directionality\;[\%]=\left[Max\left(∆\right)\frac{ABS\left\{r\left(p,q+∆\right)-r\left(q,p+∆\right)\right\}}{r\left(p,q+∆\right)+r\left(q,p+∆\right)}\cdot 2\right]$$

**Figure 4:**
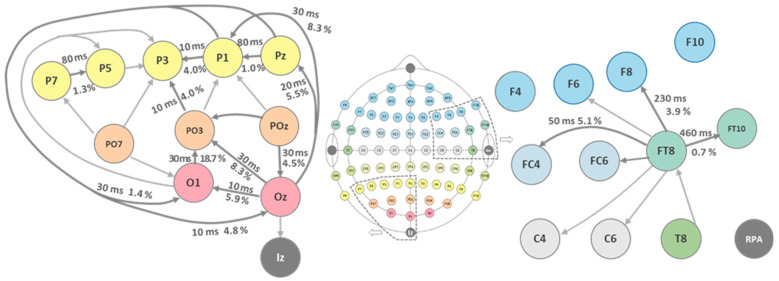
Directionalities of the EEG correlations depicted for the visual and auditory cortices. The percentage values denote the maximum differences [%] together with the latencies [ms] of those maxima. The informational flux is yielded in a correct way. So do the latencies, which are short for the more primary areas and become prolonged for the more associative spots. The auditory system shows prolonged maximum directionality latencies when compared to the visual system.

This calculation was performed for all signal pairings over the full 8 min 50 s EEG recording time. The results of these calculations are listed in Table 2. The directionality values lie in the range of a few percent up to 23%. Moreover, from both the latency data and source identification, a sound picture arises, as can be seen (Figure 4) for the occipital visual and temporal auditory lobe EEG data.

**Figure 5:**
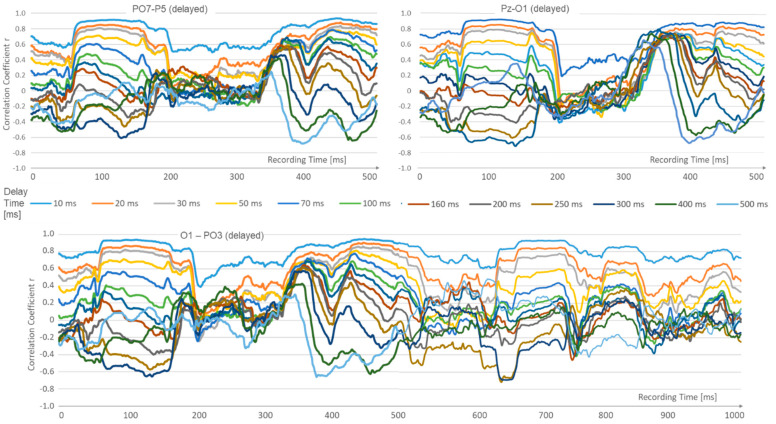
Correlation analysis over several spots and longer times reveals phases of strong short correlations, which alternate with periods where the longer delays gain strength.

Different epochs change in the recorded data: Those with high correlation, decreasing from short latencies to longer ones, are contrasted with others that show a quasi-reversal of latency. Here, the correlation collapses at short latencies, while the longer latencies up to 500 ms become increasingly positive. This epoch change occurs in periods of approximately 280 ms. See Figure 5.

## 4. Discussion

This case study addressed the hypothesis of whether EEG data allow the analysis of delays and identify directionality from surface recordings. We obtained results that EEG data can be successfully correlated, which, on the one hand, is in line with the results of Tang [36] and Bruns [54]. On the other hand, we now have numerical latency values and a practical confidence measure that can substantiate further modeling. Different latencies were calculated from recordings over the visual and auditory cortex, with shorter or longer latencies at the confidence threshold. Results are equally reflected in daily clinical experience with evoked potentials [1,46] and underline the validity of the approach taken. In particular, this is achieved from quasi-online data, since there was no stimulus-response synchronization.

The correlation of the EEG electrode data of the nearest and second-nearest neighbors shows a transmitter–receiver structure in the range of 10%, which is consistent with the state-of-the-art knowledge about primary, secondary, and associative fields [31]. These results indicate that neuronal functional structures are involved in the formation of EEG signals and that transmission does not only occur through the white matter [52,55].

The fact that it is possible to identify epochs of stable states of delay mapping on the basis of EEG latency correlation data may not appear to be new. The correlation dominance of short latency (10–70 ms) transitions into states with positive correlations of long latencies (200–500 ms), with short latencies hardly correlated at all, seems to come close to existing connectivity examinations [31,32]. However, the fact that correlation values are going hand in hand with numerical directionality evaluations is a new and original insight. The periods of these epochs are in the range of 280 ms, which is reminiscent of alpha wave periods and generalized brain states, as examined, for example, by Kringelbach and Deco [3,56]. Further work should acquire more data and compare results with existing connectivity methods, such as those described by Wendling [31], the Granger causality, or the multitude of DTFs [57,58,59].

In general, the results point to various closed-loop circuits. We estimate and identify the delays in these control loops during our correlation methodology. These should eventually be included in future modeling [12]. Overall, the proposed model is consistent with the ongoing development of classifiers for ictus detection, as reviewed by Dümpelmann [8] and Mégevand [1], as well as with stimulus localization.

## 5. Conclusions

Having discussed the previous findings well, we suppose that EEG- or ECoG-based source localization algorithms could be favorable if they add, for instance, propagational terms to dipole localization. They could use the following:

(a) Linearly simplified neural signaling (weighted signal speed and transition delays) for small-area, underlying source localizations;

(b) A set of “brain state models” according to a) that merge into one another;

(c) An adaptive, machine-learning mechanism that develops those brain states (within recurring 200–300 msec sequences) and recognizes those states.

The present results encourage the extension of the very powerful methods of epilleptogenic focus localization by kESI and kCSD to include numerical and temporal progression components. This would imply mathematically separating signals that are more likely to be attributed to instantaneous field propagation from those signal components that appear to be neuronally delayed. This could be achieved by continuous correlation and directionality analysis with a 300 msec time window. The resulting refined dipole localization could then be validated with triangulation calculations and possibly refined.

## Figures and Tables

**Table 1:** Correlation overview of 34 EEG channels from the visual and auditory cortex. Almost all correlations start from r > 0.5. Of the 34 parings, 18 reached the r = 0.7 threshold with latencies of 10 ms and longer.

**Locked Channel**	Iz	Oz	Oz	Oz	O1	PO3	POz	POz	PO3	PO3	O1	PO7	PO7	PO3	PO7	P7	P5
**Delayed Channel**	Oz	O1	POz	PO3	PO3	POz	Pz	P1	P1	P3	PO7	P7	P5	PO7	P3	P5	P3
**Corr. Coeff. @ 10 ms**	0.58	0.7	0.75	0.76	0.75	0.76	0.77	0.66	0.63	0.71	0.49	0.7	0.83	0.59	0.59	0.81	0.67
**Latency @r = 0.7/ms**	0	10	30	30	30	30	30	0	0	10	0	0	80	0	0	80	0
**Locked Channel**	P3	P1	P7	P5	P3	P1	Pz	Pz	P1	FT10	FT8	FT8	FT8	C6	FT8	FC4	FT8
**Delayed Channel**	P1	Pz	O1	O1	O1	O1	O1	Oz	Oz	FT8	F8	FC6	F6	FT8	T8	FT8	C4
**Corr. Coeff. @ 10 ms**	0.71	0.83	0.78	0.64	0.57	0.75	0.71	0.73	0.76	0.89	0.8	0.69	0.29	0.69	0.5	0.74	0.64
**Latency @r = 0.7/ms**	10	80	60	0	0	30	10	20	30	460	230	0	0	0	0	50	0

**Table 2:** Directionality and source identification calculated from EEG correlation data.

**Locked Channel**	Iz	Oz	Oz	Oz	O1	PO3	POz	POz	PO3	PO3	O1	PO7	PO7	PO3	PO7	P7	P5
**Delayed Channel**	Oz	O1	POz	PO3	PO3	POz	Pz	P1	P1	P3	PO7	P7	P5	PO7	P3	P5	P3
**Directionality [%]**	6.8	5.9	4.5	8.3	19.7	4.3	5.9	8.9	4.4	4.0	7.6	6.9	2.6	2.7	8.0	1.3	10.6
**Source**	Oz	Oz	POz	Oz	O1	POz	POz	POz	PO3	PO3	PO7	PO7	PO7	PO3	PO7	P7	P5
**Locked Channel**	P3	P1	P7	P5	P3	P1	Pz	Pz	P1	FT10	FT8	FT8	FT8	C6	FT8	FC4	FT8
**Delayed Channel**	P1	Pz	O1	O1	O1	O1	O1	Oz	Oz	FT8	F8	FC6	F6	FT8	T8	FT8	C4
**Directionality [%]**	4.0	1.0	2.3	1.5	10.4	1.4	2.7	5.5	8.3	0.7	3.9	3.4	23.5	3.8	1.9	5.1	12.6
**Source**	P1	Pz	O1	O1	O1	P1	Pz	Oz	Oz	FT9	FT8	FT8	FT8	FT8	T8	FT8	FT8

## Data Availability

Data will be posted upon publication approval.
